# A cross-sectional study of factors associated with COVID-19 testing among people who inject drugs: missed opportunities for reaching those most at risk

**DOI:** 10.1186/s12889-022-13273-y

**Published:** 2022-04-27

**Authors:** Samantha Yeager, Daniela Abramovitz, Alicia Yolanda Harvey-Vera, Carlos F. Vera, Angel Blake Algarin, Laramie Rae Smith, Gudelia Rangel, Irina Artamonova, Thomas Leroy Patterson, Angela Robertson Bazzi, Emma L. Brugman, Steffanie Ann Strathdee

**Affiliations:** 1 Division of Infectious Diseases and Global Public Health, Department of Medicine, University of California San Diego, 9500 Gilman Drive, Mail Code 0507, La Jolla, CA 92093-0507 USA; 2 Universidad Xochicalco, Facultad de Medicina, Campus Tijuana, Rampa Yumalinda 4850, Chapultepec Alamar, 22110 Tijuana, B.C. Mexico; 3 United States-Mexico Border Health Commission, Centenario 10851, Obrera, 22320 Tijuana, B.C. Mexico; 4 Departmento de Estudios de Población, El Colegio de la Frontera Norte, 22560 Tijuana, B.C. Mexico; 5 Department of Psychiatry, University of California San Diego, 9500 Gilman Drive, Mail Code 0960, La Jolla, CA 92093-0960 USA; 6 Herbert Wertheim School of Public Health, University of California San Diego, 9500 Gilman Drive Mail Code 0725, La Jolla, CA 92093-0725 USA

**Keywords:** COVID-19, SARS-CoV-2, Substance use, Testing, substance use treatment

## Abstract

**Background:**

People who inject drugs (PWID) are vulnerable to SARS-CoV-2 infection. We examined correlates of COVID-19 testing among PWID in the U.S.-Mexico border region and described encounters with services representing potential opportunities (i.e., ‘touchpoints’) where COVID-19 testing could have been offered.

**Methods:**

Between October, 2020 and September, 2021, participants aged ≥18 years from San Diego, California, USA and Tijuana, Baja California, Mexico who injected drugs within the last month completed surveys and SARS-CoV-2, HIV, and HCV serologic testing. Logistic regression identified factors associated with COVID-19 testing including potential touchpoints, comorbidities and COVID-19 related misinformation and disinformation.

**Results:**

Of 583 PWID, 30.5% previously had a COVID-19 test. Of 172 PWID who tested SARS-CoV-2 seropositive (30.1%), 50.3% encountered at least one touchpoint where COVID-19 testing could have been offered within the prior six months. Factors independently associated with at least two fold higher odds of COVID-19 testing were living in San Diego, recent incarceration, receiving substance use treatment, and experiencing ≥1 chronic health condition. Homelessness, having received ≥1 dose of COVID-19 vaccine, and having a HIV or HCV test since the COVID-19 epidemic began were also independently associated with having had a prior COVID-19 test.

**Conclusion:**

We identified several factors independently associated with COVID-19 testing and multiple touchpoints where COVID-19 testing could be scaled up for PWID, such as SUD treatment programs and syringe service programs. Integrated health services are needed to improve access to rapid, free COVID-19 testing in this vulnerable population.

## Background

Testing for SARS-CoV-2 infection is critical to identify cases who require quarantine and contact tracing, as well as treatment and supportive housing. Within the United States (U.S.), COVID-19 testing based on polymerase chain reaction (PCR) was made available at community clinics, pharmacies and laboratories since early in the pandemic [1]. However, PCR tests are expensive for those without health insurance as many sites do not provide free testing services, and turn-around times for results can take days. The nation’s first rapid at-home COVID-19 testing kit received emergency-use authorization in October, 2020 [2], but was not widely available in the U.S. for several months and often only available for a fee. In Mexico, free COVID-19 PCR tests are available at designated testing facilities or “fever clinics” for qualifying (i.e., symptomatic) individuals, and at private hospitals, laboratories or clinics for a fee [3, 4]. Despite efforts to increase the accessibility of COVID-19 testing, utilization remains low among racial/ethnic minorities and economically disadvantaged populations due to social and structural barriers related to health insurance, availability of testing sites, language, transportation, and misinformation [5–9].

COVID-19 testing misinformation (i.e., inaccurate information shared without malicious intent) and disinformation (i.e., deliberate spread of false information) have been negating efforts to increase COVID testing behaviors [10]. COVID-19 misinformation is a prominent barrier to testing among African American and Latinx communities [6, 11]. Research within the U.S. and United Kingdom has also highlighted connections between COVID-19 disinformation (e.g., conspiracy theories) and lower engagement in preventive behaviors (e.g., handwashing, mask wearing, and social distancing) and vaccination [12, 13]. In a previous analysis, COVID-19 disinformation was significantly associated with COVID-19 vaccine hesitancy among people who inject drugs (PWID) in the U.S.-Mexico border region [13]. However, it remains unknown if COVID-19 misinformation or disinformation impacts COVID-19 testing utilization among PWID.

Due to their high prevalence of chronic diseases [14], homelessness [15, 16], food insecurity [17], frequent incarceration [18, 19], and behavioral risk factors (e.g., engaging in sex work, sharing needles with others) [18], PWID are at elevated risk for SARS-CoV-2 infection and developing severe symptoms [14, 18, 20]. PWID often underutilize healthcare services due to stigma, discrimination, medical mistrust [21, 22], and mistreatment [15, 23, 24]. However, some PWID could receive COVID-19 testing through intersecting venues or touchpoints including substance use disorder (SUD) treatment programs, syringe service programs (SSPs), emergency rooms, and jail/prisons [25–28].

We identified correlates of COVID-19 testing and described interactions with services or venues where COVID-19 testing could have been offered, drawing from literature on overdose prevention ‘touchpoints’ within the healthcare system [29]. We hypothesized that socio-structural determinants (e.g., food/housing insecurity, Latinx ethnicity), and COVID-19 disinformation and misinformation would be associated with less COVID-19 testing. We also hypothesized that PWID with chronic health conditions and those who had recently been incarcerated or received health care services would be more likely to have had a COVID-19 test.

## Methods

### Participants and eligibility

Between October 28, 2020 and September 10, 2021, people aged ≥18 or older who injected drugs within the last month and lived in San Diego County or Tijuana were recruited through street outreach, as previously described [18]. Recruitment took place using a recreational vehicle whereby potential participants were approached by outreach workers in various locations, such as on the street, parks, shelters, motels, river canyons and vacant lots. All participants provided written informed consent. Protocols were approved by the Human Research Protection Program at the University of California San Diego and institutional review board at Xochicalco University in Tijuana. The study was conducted in accordance with The Code of Ethics of the World Medical Association (Declaration of Helsinki).

### Survey measures

Participants underwent face-to-face interviewer-administered questionnaires using computer assisted personal interviews. To reduce participant burden, some survey items were administered at baseline and approximately one week later for which they were compensated $20 USD and $10 USD, respectively. Surveys assessed socio-demographics, number of hours spent on the street on a typical day over the past 6 months (including looking for drugs, obtaining money or sleeping), injection and non-injection use of specific drugs (ever and in the last six months), chronic health problems (e.g., diabetes, asthma, hypertension), food insecurity [30], COVID-19 experiences (negative income impact/food insecurity, knows someone who died from COVID), COVID-19 exposures, and protective behaviors (e.g., social distancing, masking). Generalized anxiety disorder symptoms were assessed through the GAD-7 [31, 32], which demonstrated high internal reliability in the current study (α = 0.93, ω = 0.78). Perceived threat of COVID-19 was assessed by asking how worried they were about getting COVID-19 (or getting it again) on a ten point scale [33].

Participants were asked if they had ever received a COVID-19 test, and if so, to specify the date, location and result (if known). We also inquired about encounters with potential COVID-19 testing touchpoints (i.e., where COVID-19 testing could have been offered) in the last six months [29]. These included being enrolled in a SUD treatment program, having been incarcerated, sleeping in a shelter, using a SSP, having an overdose, or having been tested for HIV or HCV since the COVID-19 epidemic began.

To assess COVID-19 misinformation, we presented participants with seven statements about SARS-CoV-2 transmission, severity, immunity, symptoms, treatments and vaccines and asked them to classify each statement as “True”, “False,” or “Unsure” [13]. These included the following: (1) COVID-19 cannot be easily spread from one person to another; (2) many thousands of people have not died from COVID-19; (3) most people are immune to COVID-19; (4) you can tell someone has COVID-19 from looking at them; (5) there are treatments that can cure COVID-19; and (6) COVID-19 is about as dangerous as having the flu. We then created a binary variable for each statement indicating whether the participant was misinformed or not.

COVID-19 disinformation was assessed through a six-item scale including conspiracy theory items as previously described [13]. These included “COVID-19 was created by the pharmaceutical industry” or “the Chinese government”, “childhood vaccines cause autism” [34], as well as three additional items: “COVID-19 vaccines include a tracking device”, “alter DNA”, and “COVID-19 vaccines offered to ‘people like me’ are not as safe”. We dichotomized responses to indicate endorsement of disinformation (“True” and “Unsure”) or not (“False”) and summed them into a total score ranging from 0 to 6. Cronbach’s alpha and McDonald’s omega were 0.78. The mean inter-item correlation value was 0.31, which indicates optimal internal consistency [35].

### SARS-CoV-2 antibody detection

Blood samples were collected by venipuncture. Serums were batched and tested weekly by Genalyte® (San Diego, CA), using their Maverick™ Multi-Antigen Serology Panel [36] that detects IgG and IgM antibodies to five SARS-CoV-2 antigens. A machine learning algorithm was used to call results using the Random Forest Ensemble method with 3000 decision trees [37].

### HIV and HCV serology

Rapid HIV and HCV tests were conducted using the Miriad® HIV/HCV Antibody InTec Rapid Anti-HCV Test (Avantor, Radnor, PA). Reactive and indeterminate tests underwent a second rapid test with Oraquick® HIV or Oraquick® HCV, respectively (Orasure, Bethlehem, PA) and were confirmed by Western Blot at the UC San Diego Centers for AIDS Research.

### Statistical analysis

The outcome for this analysis was reporting having had a COVID-19 test prior to joining the study (yes/no). Characteristics of participants who had a COVID-19 test versus those who had not were compared using Mann-Whitney U tests for continuous variables and Chi-square tests or Fisher’s exact tests for categorical variables. Univariate and multivariable logistic regressions with robust standard error estimation via generalized estimating equations were performed to identify factors associated with COVID-19 testing.

Variables attaining significance at α = 0.10 in univariate regression models were considered candidates for inclusion in multivariable models, using Hosmer and Lemeshow’s purposeful selection of variables approach [38] to arrive at a final model. Variables were retained in the final multivariable model based on statistical significance and relationships among potential predictors (e.g., correlations, confounding, and interactions). Since availability of COVID-19 testing may have changed during the 11 month study period, we included a linear term representing the time that had elapsed since the interview. Since there was no interaction by site (i.e., residence in San Diego versus Tijuana), we did not stratify by place of residence. All statistical analyses were conducted using SAS, version 9.4.

## Results

### Sample characteristics and COVID-19 testing history

A total of 583 participants who completed baseline and supplemental interviews and responded to questions about COVID-19 testing history were included in this analysis. The majority identified as male (74.3%) and Hispanic, Latinx, or Mexican (73.6%) and 37.7% had completed high school or its equivalent. By design, approximately half (58.7%) resided in San Diego County (Table 1). Mean age was 43 years (standard deviation [SD]: 11).

**Table 1:** Characteristics Associated with COVID-19 Testing among PWID in San Diego, CA and Tijuana, Mexico (*N* = 583)

Baseline Characteristics	Tested prior to joining the study ***N*** = 178	NOT tested prior to joining the study ***N*** = 405	Total ***N*** = 583	***p***-value
***Sociodemographic Factors***				
Male	130(73.0%)	303(74.8%)	433(74.3%)	0.65
Mean Age (SD)	43.4(11.1)	43.2(10.4)	43.2(10.6)	0.87
Hispanic/Latinx/Mexican	96(53.9%)	333(82.2%)	429(73.6%)	<.001
Speaks English	160(89.9%)	258(63.7%)	418(71.7%)	<.001
Born in the US	142(79.8%)	152(37.5%)	294(50.4%)	<.001
Primary residence in San Diego	149(83.7%)	193(47.7%)	342(58.7%)	<.001
Homeless*	100(56.2%)	155(38.3%)	255(43.7%)	<.001
Completed high school or its equivalent	100(56.2%)	120(29.6%)	220(37.7%)	<.001
Average monthly income < 500 USD	78(43.8%)	252(62.2%)	330(56.6%)	<.001
***Behavioral Factors***				
Mean # of hours spent on the street (SD)*	17.0(7.9)	14.7(7.2)	15.4(7.5)	0.001
Engaged in sex work*	20(11.2%)	56(13.8%)	76(13.0%)	0.39
***Substance Use Factors***				
Smoked/snorted/inhaled/vaped methamphetamine*	132(74.2%)	236(58.3%)	368(63.1%)	<.001
Smoked/snorted/inhaled cocaine*	36(20.2%)	29(7.2%)	65(11.1%)	<.001
Smoked/snorted/inhaled/vaped fentanyl*	63(35.4%)	45(11.1%)	108(18.5%)	<.001
Smoked/snorted/inhaled heroin*	69(38.8%)	87(21.5%)	156(26.8%)	<.001
Injected methamphetamine*	106(59.6%)	170(42.0%)	276(47.3%)	<.001
Injected fentanyl*	55(30.9%)	63(15.6%)	118(20.2%)	<.001
Injected heroin*	153(86.0%)	358(88.4%)	511(87.7%)	0.41
Mean # of years of injection drug use (SD)	21.2(12.7)	20.6(12.0)	20.8(12.2)	0.75
Mean # of times injected drugs per day (SD)*	2.2(1.4)	2.5(1.6)	2.4(1.5)	0.01
***Mental Health & Attitudinal Factors***				
Mean GAD-7 anxiety scale (SD)	14.2(6.4)	12.8(6.0)	13.2(6.1)	0.01
Mean for: On a scale of 1 to 10, how worried are you of getting COVID-19 (or getting it again)(SD)	4.5(3.3)	5.1(3.0)	4.9(3.1)	0.01
***COVID-19 Misinformation and Disinformation***				
COVID-19 Misinformation: Does NOT think that the virus that causes COVID-19 can be easily spread from one person to another ^Y3^	33(20.9%)	90(23.3%)	123(22.6%)	0.54
COVID-19 Misinformation: Does NOT think that many thousands of people have died from COVID-19^Y3^	17(10.8%)	59(15.3%)	76(14.0%)	0.17
COVID-19 Misinformation: Thinks that most people already have immunity to COVID-19 ^Y3^	101(63.9%)	257(66.6%)	358(65.8%)	0.55
COVID-19 Misinformation: Thinks that you can tell someone has COVID-19 by looking at them ^Y3^	35(22.2%)	82(21.2%)	117(21.5%)	0.81
COVID-19 Misinformation: Thinks that there are effective treatments for COVID-19 that can cure most people ^Y3^	111(70.3%)	302(78.2%)	413(75.9%)	0.05
COVID-19 Misinformation: Thinks that having COVID-19 is about as dangerous as having the flu ^Y3^	100(63.3%)	219(56.7%)	319(58.6%)	0.16
COVID-19 Disinformation: Thinks the pharmaceutical industry created the COVID-19 virus ^Y3^	76(48.4%)	166(43.0%)	242(44.6%)	0.25
COVID-19 Disinformation: Thinks COVID-19 was created by the Chinese government as a biological weapon ^Y3^	95(60.5%)	191(49.5%)	286(52.7%)	0.02
COVID-19 Disinformation: Thinks the vaccines given to children for diseases like measles and mumps cause autism ^Y3^	99(62.7%)	218(56.5%)	317(58.3%)	0.18
COVID-19 Disinformation: Thinks that COVID vaccines being offered to ‘people like me’ are not as safe as other COVID vaccines ^Y3^	64(40.5%)	116(30.1%)	180(33.1%)	0.02
COVID-19 Disinformation: Thinks that COVID vaccines include a tracking device ^Y3^	45(28.5%)	98(25.4%)	143(26.3%)	0.46
COVID-19 Disinformation: Thinks that some COVID vaccines could change their DNA ^Y3^	56(35.4%)	93(24.1%)	149(27.4%)	0.01
***Health-Related Factors***				
Ever had a flu vaccine ^Y2^	97(61.4%)	142(37.0%)	239(44.1%)	<.001
Tested HIV+	9(5.1%)	37(9.1%)	46(7.9%)	0.10
Tested HCV+	79(44.9%)	147(36.3%)	226(38.9%)	0.05
Has at least one chronic illness (excluding seasonal allergies and acne/skin problems)	91(51.1%)	120(29.6%)	211(36.2%)	<.001
Mean # of chronic conditions (excluding seasonal allergies and acne/skin problems) (SD)	0.9(1.3)	0.5(1.0)	0.6(1.1)	<.001
***COVID-19-Related Factors***				
Income worse since COVID began ^Y1^	107(60.8%)	289(72.4%)	396(68.9%)	0.01
Low/very low food security since COVID began	137(77.0%)	335(82.7%)	472(81.0%)	0.10
Exposed to someone with COVID-19	21(11.8%)	13(3.2%)	34(5.8%)	<.001
Knows someone who died from COVID-19 ^Y5^	58(36.5%)	112(28.9%)	170(31.1%)	0.08
Reported being vaccinated for COVID-19	30(16.9%)	44(10.9%)	74(12.7%)	0.06
Tested SARS-CoV-2 seropositive ^Y6^	50(29.4%)	122(30.4%)	172(30.1%)	0.37
Practiced Social Distancing	86(48.3%)	82(20.2%)	168(28.8%)	<.001
Wore a face mask	152(85.4%)	314(77.5%)	466(79.9%)	0.03
Most important source of COVID-19-related information: Friends ^Y4^	48(31.6%)	199(52.2%)	247(46.3%)	<.001
Most important source of COVID-19-related information: Doctors/health professionals ^Y4^	22(14.5%)	12(3.1%)	34 (6.4%)	<.001
Most important source of COVID-19-related information: Social media ^Y4^	30(19.7%)	33(8.7%)	63(11.8%)	<.001
***Touchpoints Representing Opportunities for COVID-19 Testing***				
Incarcerated*	32(18.0%)	26(6.4%)	58(10.0%)	<.001
Slept in a shelter/welfare residence*	25(14.0%)	17(4.2%)	42(7.2%)	<.001
Overdose*	37(20.9%)	50(12.3%)	87(14.9%)	0.01
Tested for HIV or HCV post-COVID	91(52.6%)	139(34.6%)	230(40.0%)	<.001
Has been enrolled in a drug treatment program*	29(16.3%)	21(5.2%)	50(8.6%)	<.001
Has been enrolled in a methadone or buprenorphine program*	22(12.4%)	15(3.7%)	37(6.3%)	<.001
Attended a syringe service program*	8(4.5%)	6(1.5%)	14(2.4%)	0.03

*Past 6 months

Missing values: ^Y1^ *n* = 8, ^Y2^*n* = 41; ^Y3^*n* = 39; ^Y4^*n* = 50; ^Y5^*n* = 37; ^Y6^*n* = 12

*Note: All the n (%) represent the affirmative response to the binary variables*

In the past six months, 43.7% of participants were homeless. The majority injected heroin (87.7%), methamphetamine (47.3%) or fentanyl (20.2%) in the last six months. Most had also smoked, snorted or inhaled or methamphetamine (63.1%), heroin (26.8%), fentanyl (18.5%) or cocaine (11.1%) in the last six months. Over one third tested HCV-seropositive (38.9%), 7.9% tested HIV-seropositive and 36.2% reported at least one other chronic health condition (e.g., diabetes, hypertension).

Overall, 178 participants (30.5%) reported that they previously had a COVID-19 test. Of 105 participants who were asked the location of their COVID-19 test in a supplemental survey, the most common testing locations were community clinics (including mobile clinics and health fairs; 55.2%), hospitals (14.3%), doctors’ offices (14.3%), jail/prison/detention centers (10.5%), SSPs (5.7%), SUD treatment clinics (2.9%), and pharmacies (1.9%).

Considering potential touchpoints for COVID-19 testing, 40% had received an HIV or HCV test outside of the study since the COVID-19 epidemic began. In the last 6 months, 15% had an overdose, 10% had been incarcerated, 7.2% slept in a shelter or a welfare residence, 8.6% had visited a SUD treatment program and 2.4% had used a SSP. Of the 405 participants who had not had a prior COVID-19 test, almost half (46%) reported at least one touchpoint encounter where COVID-19 testing could have been offered. Furthermore, of 571 participants who provided blood samples for SARS-CoV-2 serology and who tested seropositive in our study (*N* = 172, 30.1%), 70.9% had not previously had a COVID-19 test and 50.3% had encounters with at least one touchpoint where COVID-19 testing could have been offered.

### Factors associated with COVID-19 testing in bivariate analysis

Sociodemographic Factors. Compared to those who had not had a prior COVID-19 test, those who had been tested were more likely to be living in San Diego County (versus Tijuana) and were less likely to identify as Hispanic, Latinx or Mexican. COVID-19 testing was positively associated with having completed high school or its equivalent and being homeless in the last six months (Tables 1 and 2).

**Table 2:** Factors associated with SARS-CoV-2 testing in Tijuana and San Diego

Baseline Characteristics	Univariate OR (95% CI)
***Sociodemographic Factors***	
Male^P^	0.91 (0.61,1.36)
Age^P^	1.00 (0.99,1.02)
Hispanic/Latinx/Mexican	0.25 (0.17,0.37)
Speaks English	5.06 (2.99,8.58)
Born in the US	6.57 (4.33,9.97)
Primary residence in San Diego	5.64 (3.62,8.79)
Homeless*	2.07 (1.45,2.96)
Completed high school or its equivalent	5.64 (3.62,8.79)
Monthly income < 500 USD	0.47 (0.33,0.68)
***Behavioral Factors***	
# of hours spent on the street on a typical day*	1.04 (1.02,1.07)
Engaged in sex work*^P^	0.79 (0.46,1.36)
***Substance Use Factors***	
Smoked/snorted/inhaled/vaped methamphetamine*	2.05 (1.39,3.03)
Smoked/snorted/inhaled cocaine*	3.29 (1.94,5.56)
Smoked/snorted/inhaled/vaped fentanyl*	4.38 (2.83,6.78)
Smoked/snorted/inhaled/vaped heroin*	2.31 (1.58,3.40)
Injected methamphetamine*	2.04 (1.42,2.91)
Injected fentanyl*	2.43 (1.60,3.68)
Injected heroin*^P^	0.80 (0.48,1.35)
Years of injection drug use^P^	1.00 (0.99,1.02)
# Times injected drugs per day	0.87 (0.78,0.98)
***Mental Health and Attitudinal Factors***	
GAD-7 anxiety scale	1.04 (1.01,1.07)
On a scale of 1 to 10, how worried are you of getting COVID-19 (or getting it again)	0.94 (0.88,0.99)
***COVID-19 Misinformation and Disinformation***	
COVID-19 Misinformation: Does NOT think the virus that causes COVID-19 can be easily spread from one person to another^Y3P^	0.87 (0.55,1.36)
COVID-19 Misinformation: Does NOT think that many thousands of people have died from COVID-19^Y3P^	0.67 (0.38,1.19)
COVID-19 Misinformation: Thinks that most people already have immunity to COVID-19^Y3P^	0.89 (0.60,1.31)
COVID-19 Misinformation: Thinks that you can tell someone has COVID-19 by looking at them^Y3P^	1.05 (0.67,1.65)
COVID-19 Misinformation: Thinks that there are effective treatments for COVID-19 that can cure most people^Y3^	0.66 (0.43,1.00)
COVID-19 Misinformation: Thinks that having COVID-19 is about as dangerous as having the flu^Y3P^	1.31 (0.90,1.92)
COVID-19 Disinformation: Thinks the pharmaceutical industry created the COVID-19 virus ^Y3 P^	1.24 (0.86,1.80)
COVID-19 Disinformation: Thinks COVID-19 was created by the Chinese government as a biological weapon ^Y3^	1.56 (1.07,2.28)
COVID-19 Disinformation: Thinks the vaccines given to children for diseases like measles and mumps cause autism ^Y3 P^	1.29 (0.88,1.89)
COVID-19 Disinformation: Thinks the pharmaceutical industry created the COVID-19 virus ^Y3 P^	1.24 (0.86,1.80)
COVID-19 Disinformation: Thinks that COVID vaccines being offered to ‘people like me’ are not as safe as other COVID vaccines ^Y3^	1.58 (1.08,2.33)
COVID-19 Disinformation: Thinks that COVID vaccines include a tracking device ^Y3 P^	1.17 (0.77,1.77)
COVID-19 Disinformation: Thinks that some COVID vaccines could change their DNA ^Y3^	1.73 (1.16,2.58)
***Health-Related Factors***	
Ever had a flu vaccine^Y2^	2.71 (1.85,3.97)
Tested HIV+	0.53 (0.25,1.12)
Tested HCV+	1.43 (1.00,2.05)
Has at least one chronic condition (excluding seasonal allergies and acne/skin problems)	2.48 (1.73,3.57)
# of chronic conditions (excluding seasonal allergies and acne/skin problems	1.40 (1.19,1.65)
***COVID-19-Related Factors***	
Income worse since COVID began^Y1^	0.59 (0.41,0.86)
Low or very low food security since COVID began	0.70 (0.45,1.08)
Exposed to someone with COVID-19	4.03 (1.97,8.25)
Knows someone who died of COVID-19^Y5^	1.41 (0.95,2.08)
Reported being vaccinated for COVID-19	1.66 (1.01,2.75)
Tested SARS-CoV-2 seropositive^Y6P^	1.05 (0.83,1.33)
***COVID-19 Protective Behaviors***	
Social Distancing	3.68 (2.52,5.39)
Wore face mask	1.69 (1.05,2.73)
Most important source of COVID-19-related information: Friends^Y4^	0.42 (0.28,0.63)
Most important source of COVID-19-related information: Doctors/health professionals^Y4^	5.20 (2.50,10.8)
Most important source of COVID-19-related information: Social media^Y4^	2.59 (1.52,4.43)
***Touchpoint Opportunities for COVID-19 Testing***	
Incarcerated*	3.19 (1.84,5.53)
Slept in a shelter/welfare residence*	3.73(1.96, 7.10)
Overdose*	1.88 (1.18,3.00)
Tested for HIV or HCV post-COVID	2.10 (1.46,3.02)
Has been enrolled in a drug treatment program*	3.56 (1.97,6.44)
Has been enrolled in a methadone or buprenorphine program*	3.67 (1.85,7.25)
Attended a syringe service program*	3.13 (1.07,9.16)

*Past 6 months; Missing values: ^Y1^ *n* = 8, ^Y2^*n* = 41; ^Y3^*n* = 39; ^Y4^*n* = 50; ^Y5^*n* = 37; ^Y6^*n* = 12; ^P^P-value> 0.10 (all others <=0.10)

Behavioral and Substance Use Factors. Behaviors significantly associated with higher odds of COVID-19 testing included non-injection use of fentanyl or injecting methamphetamine in the last six months and spending more time on the street.

Mental Health and Attitudinal Factors*.* Increased anxiety reflected by higher GAD-7 scores and expressing greater worry about COVID-19 were associated with higher odds of COVID-19 testing.

COVID-19 Misinformation and Disinformation*.* Endorsing most statements reflecting COVID-19 misinformation or disinformation were not significantly associated with a lower odds of COVID-19 testing with the exception of believing that the coronavirus was created by the Chinese government as a biological weapon.

Health-related Factors. Having diabetes, at least one chronic condition, ever having had a flu vaccine and testing HCV or HIV seropositive were significantly associated with COVID-19 testing.

COVID-19 Related Factors and Protective Behaviors. Using facemasks, practicing social distancing, having received at least one COVID-19 vaccine dose, and having been exposed to someone with COVID-19 were significantly associated with COVID-19 testing. Having primarily obtaining their COVID-19 information from social media or health providers was significantly associated with higher odds of COVID-19 testing, whereas obtaining most of their COVID-19 information from friends was inversely associated with COVID-19 testing.

Touchpoints for COVID-19 Testing. Having been incarcerated, overdosed, slept in a shelter, received SUD treatment or visited a SSP program in the last six months were significantly associated with higher odds of COVID-19 testing.

### Factors associated with COVID-19 testing in multivariate analysis

Factors Independently Associated with COVID-19 Testing. Our final multivariate model reflecting factors that were independently associated with COVID-19 testing while controlling for time is displayed in Table 3. Living in San Diego County (versus Tijuana), and having been incarcerated or enrolled in a SUD treatment program in the last six months were independently associated with COVID-19 testing. Having at least one chronic condition, receiving at least one COVID-19 vaccine dose, having been homeless or using fentanyl by means other than injection in the last six months were also independently associated with having had a COVID-19 test. Having been tested for HIV or HCV since the COVID-19 epidemic began remained marginally associated with COVID-19 testing.

**Table 3:** Factors Independently Associated with COVID-19 Testing among PWID in San Diego, CA and Tijuana, Mexico

Baseline Characteristics	Adjusted OR (95% CI)	Pr > ChiSq
Primary residence in San Diego	4.52 (2.69, 7.60)	<.001
Incarcerated^*^	2.72 (1.29, 5.73)	.009
Got tested for HIV or HCV since COVID-19 began	1.52 (0.97, 2.38)	.07
Homeless^*^	1.77 (1.12, 2.77)	.01
Reported having at least one COVID-19 vaccine dose	1.97 (1.03, 3.79)	.04
Smoked/snorted/inhaled/vaped fentanyl^*^	1.83 (1.04, 3.20)	.04
Has at least one chronic health condition	2.66(1.68, 4.22)	<.001
Enrolled in a SUD treatment program^*^	2.41 (1.12, 5.21)	.03
Months that elapsed since the supplemental interview^¥^	1.10 (1.00, 1.20)	.05

^*^Past 6 months; ^¥^Per one unit increase

## Discussion

We identified several factors that were independently associated with COVID-19 testing among PWID in the Mexico-US border region, as well as multiple touchpoints where COVID-19 testing could have been offered. Although SARS-CoV-2 prevalence among PWID in San Diego County and Tijuana is higher than that of the general population in either city [18], less than one third of our sample had ever been tested for COVID-19. Of concern, over two thirds of participants who tested SARS-CoV-2 seropositive in our study had not previously had a COVID-19 test and half reported at least one missed opportunity for testing. Our findings are consistent with a study of individuals who currently and formerly used drugs in Baltimore, Maryland, which found that only 13% had received a COVID-19 test in the first quarter of the pandemic [16]. Similarly, in a study of PWID in England and Northern Ireland conducted in 2020, only 22% had ever had a COVID-19 test [39]. These findings have implications for improving service delivery for this vulnerable population, as well as broader efforts to reduce SARS-CoV-2 transmission and morbidity and mortality in marginalized communities.

An encouraging finding was that PWID who reported receiving SUD treatment were more likely to have been tested for COVID-19. This is supported by data from a recent study of 265 clients receiving residential SUD treatment in Southern California, among whom 74% had received a COVID-19 test [26]. Although it is not clear whether or not individuals had received testing at the SUD program itself or whether SUD treatment was a marker for health-seeking behaviors, some participants did report having had a COVID-19 test at SUD treatment clinics in our study. SUD treatment programs could serve as an ideal venue for providing COVID-19 testing as well as vaccines and education to dispel myths about COVID-19 testing and vaccination. However, during the pandemic, some SUD treatment programs were suspended or only offered take-home or telemedicine services [40], potentially reducing opportunities for the provision of other services.

We also found that recent incarceration was associated with more than a two-fold higher odds of having had a COVID-19 test. Indeed, one in ten individuals who had a prior COVID-19 test reported that they obtained the test in a jail, prison or detention center. In many of these cases, COVID-19 testing may have been mandatory, since COVID-19 outbreaks have been reported in correctional facilities in California and elsewhere [41] and incarceration was independently associated with SARS-CoV-2 seropositivity among PWID in our study sample [18]. These outbreaks prompted mass SARS-CoV-2 testing in some jurisdictions [25]. A study conducted in 2020 among the U.S. Federal Bureau of Prisons found that half of the prison populace had been subjected to COVID-19 testing [42].

As expected, PWID who were already in contact with the healthcare system were more likely to have had a COVID-19 test. Specifically, those who had been tested for HIV or HCV since the COVID-19 epidemic began, and those who had received at least one COVID-19 vaccine dose were significantly more likely to have had a COVID-19 test. Furthermore, those who had at least one chronic condition were more likely to receive COVID-19 testing, which is noteworthy since individuals with co-morbidities are at greater risk of developing serious complications associated with COVID-19 [14].

Our findings that homelessness and non-injection use of fentanyl (e.g., smoked, snorted, inhaled, vaped) were both independently associated with having a COVID-19 test was surprising. However, this could be explained by concerted efforts in both San Diego and Tijuana to provide outreach to people experiencing homelessness during the COVID-19 pandemic, for example, through health fairs, mobile testing units and temporary housing. Similarly, individuals who report using fentanyl likely had greater addiction severity and may have been more likely to come into contact with community-based health providers who offered COVID-19 testing. Compared to other illicit substances, fentanyl is a highly potent drug with heightened risks for experiencing an overdose [43], which may have led to increased contact with medical settings (e.g., Emergency Departments) [44] and subsequently COVID-19 testing. These interpretations are speculative and deserve greater attention. Kral and colleagues have documented marked transitions from injection of black tar heroin to non-injection use of fentanyl in San Francisco [45] and this sub-group of substance users may be more attuned to their health.

Contrary to expectation, we did not find that Latinx ethnicity was associated with a lower odds of COVID-19 testing; however, ethnicity was highly correlated with place of residence. We did not find that COVID-19 disinformation or misinformation were significantly associated with a reduced odds of COVID-19 testing after controlling for other factors, perhaps because these measures were not specific to COVID-19 testing. Future studies may benefit by incorporating tailored measures on COVID-19 disinformation and misinformation that specifically address COVID testing which should be informed by qualitative research to more fully assess the influence of these factors on COVID-19 testing behaviors.

The lack of affordable and accessible rapid COVID-19 testing has been a major shortfall of the public health response to the pandemic in the United States, Mexico [9, 46, 47], and elsewhere. Considering that over half of our participants earned less than $500 USD per month and the prevalence of homelessness was high, it is unreasonable to expect that this population would have access to financial or transportation resources (or sufficient access to the Internet) to be able to purchase expensive at-home test kits or make and attend appointments for COVID-19 testing. To reduce high rates of morbidity and mortality due to COVID-19 and ongoing SARS-CoV-2 transmission, it is critical that infections among PWID and other vulnerable populations are detected early, especially given their high prevalence of SARS-CoV-2 infection [18]. Efforts to expand free rapid testing for PWID at venues they already access and trust are especially needed, given recent evidence suggesting that people with SUD may be more prone to breakthrough infections following vaccination due to their high prevalence of co-morbidities [48].

### Limitations

This study was limited by the cross-sectional nature of the analysis, which prevented us from drawing causal inferences. Although this was a binational study, sampling was non-random and our findings may not generalize to other PWID populations. Our reliance on self-report may have led to socially desirable responding or problems with recall. We were unable to differentiate between situations where COVID-19 testing was mandatory versus situations in which testing was voluntary and sought out by participants. For example, COVID-19 testing was likely required for participants entering correctional facilities and may also have been mandatory in some shelters. Similarly, we did not ask participants if they were required to pay for COVID-19 testing. Since both the availability and the cost of COVID-19 testing may have changed during the pandemic, we controlled for time in our analysis. It should be also noted that after study recruitment concluded, at-home tests became freely available from the U.S. government [49] in January 2022. However, access to government distributed at-home testing kits may still pose access difficulties for some populations, such as those who are unstably housed or people with low health literacy.

Some associations may not have been detected due to low statistical power. For example, the lack of an association between COVID-19 testing and attending SSPs was likely due to the small number of participants who had recently accessed these programs, since service provision for some harm reduction programs across the U. S was compromised during the pandemic [27, 50].

## Conclusions

Although SARS-CoV-2 seroprevalence among PWID in San Diego County and Tijuana was higher than the general population in their respective cities, we found that over two thirds of those seropositive for SARS-CoV-2 had never had a prior COVID-19 test. Notably, half reported at least one touchpoint encounter where they could have received COVID-19 testing but did not. Our participants were more likely to have had prior COVID-19 testing if they had received care for a comorbid health condition, had been tested for HIV or HCV, recently received treatment for SUD or if they had been incarcerated. Given the overall low level of COVID-19 testing and numerous missed opportunities for testing, our findings highlight the urgent need to improve access to free rapid COVID-19 tests in venues that PWIDs trust and routinely access. Such initiatives may also improve uptake of COVID-19 vaccines.

## Data Availability

The datasets generated and/or analysed during the current study are not publicly due to the fact that the study team is still collecting data until August 31st, 2023, but data are available from the corresponding author on reasonable request. For more information, please contact Steffanie Strathdee, Ph.D. at sstrathdee@health.ucsd.edu.

## Abbreviations

PWID: People Who Inject Drugs
SUD: Substance Use Disorder
SSP: Syringe Sharing Program
